# Three-Dimensional Printed Resin: Impact of Different Cleaning Protocols on Degree of Conversion and Tensile Bond Strength to a Composite Resin Using Various Adhesive Systems

**DOI:** 10.3390/ma16093580

**Published:** 2023-05-07

**Authors:** Valerie Lankes, Marcel Reymus, Felicitas Mayinger, Andrea Coldea, Anja Liebermann, Moritz Hoffmann, Bogna Stawarczyk

**Affiliations:** 1 Department of Prosthetic Dentistry, University Hospital, LMU Munich, 80336 Munich, Germany; felicitas.mayinger@med.uni-muenchen.de (F.M.); andrea.coldea@med.uni-muenchen.de (A.C.); moritz.hoffmann@med.uni-muenchen.de (M.H.); 2 Department of Conservative Dentistry and Peridontology, University Hospital, LMU Munich, 80336 Munich, Germany; marcel.reymus@med.uni-muenchen.de; 3 Head of the Department of Prosthetic Dentistry, Faculty of Medicine and University Hospital Cologne, 50931 Cologne, Germany; anja.lieberman@uk-koeln.de; 4 Scientific Head Dental Material Unit, Department of Prosthetic Dentistry, University Hospital, LMU Munich, 80336 Munich, Germany; bogna.stawarczyk@med.uni-muenchen.de

**Keywords:** cleaning, 3D temporary resin, degree of conversion, adhesives, tensile bond strength, failure types

## Abstract

The present investigation tested the effect of cleaning methods and adhesives on the tensile bond strength (TBS) of a resin-based composite luted to a temporary 3D printed resin. Substrates (*n*= 360) were printed using a Rapidshape D20II and cleaned with a butyldiglycol-based solution, isopropanol, or by centrifugation. Specimens were air-abraded with Al_2_O_3_ (mean particle size 50 µm) at 0.1 MPa followed by pretreatment (*n* = 30/subgroup) with: (1) Clearfil Ceramic Primer (CCP); (2) Clearfil Universal Bond (CUB); (3) Scotchbond Universal Plus (SUP) or 4. Visio.link (VL) and luted to PanaviaV5. TBS (*n* = 15/subgroup) was measured initially (24 h at 37 °C water) or after thermal cycling (10,000×, 5/55 °C). The degree of conversion (DC) for each cleaning method was determined prior and after air-abrasion. Univariate ANOVA followed by post-hoc Scheffé test was computed (*p* < 0.05). Using Ciba-Geigy tables and chi-square, failure types were analyzed. The DC values were >85% after all cleaning methods, with centrifugation showing the lowest. CCP pretreatment exhibited the lowest TBS values, with predominantly adhesive failures. The combination of CCP and centrifugation increased the TBS values (*p* < 0.001) compared to the chemical cleaning. CUB, SUP, and VL, regardless of cleaning, can increase the bond strength between the 3D printed resin and the conventional luting resin.

## 1. Introduction

In the last decade, the application of tooth-colored resin-based materials manufactured with CAD (computer-aided design)/CAM (computer-aided manufacturing) technology has increased in all fields of dentistry. Additive manufacturing (AM) supports maxillofacial surgery with surgical templates, orthodontics benefits from individualized brackets, students practice root canals treatments on printed teeth, and dental prosthetics applies printed bite splints and dentures [1]. The development of ceramic-filled hybrid materials enables the use of printed restorations as permanent fixed restorations, as they contain varying amounts of inorganic fillers (e.g., glass–ceramic fillers) in addition to a resin matrix and initiators [2]. Within AM, the most commonly used manufacturing technique is VAT polymerization, in which the liquid, light-curing resin is added to a vat of the printer and cured by a controlled supply of ultraviolet light, and, layer by layer, the object is built up on the building platform [3], requiring a subsequent cleaning of the printed objects. Chemical cleaning, especially with isopropanol, is the most used method, as such liquids are easy to obtain. Some companies have addressed the purification of 3D printed objects and launched specifically developed solutions (e.g., InovaPrint wash). Physical cleaning by using centrifugal force has shown an additional positive influence on the material quality of the printed objects [4]. For the long-term use of dental materials in the oral cavity, their biocompatibility is of utmost importance. The biocompatibility of resin-based materials essentially depends on the degree of conversion of the carbon double bonds, since non-polymerized residual monomers in the resin structure present themselves as leachable components and can go into solution in liquid media [5]. Leachable substances can trigger systemic and local immune reactions in the organism, as well as induce allergies in dentists and dental technicians [6]. In printed restorations, there are several parameters that influence the degree of conversion of carbon–carbon double bonds [7]. It has already been shown that the polymerization device Otoflash G171, which polymerizes under oxygen-free conditions using a nitrogen atmosphere, has a higher carbon double bond rate than comparable devices [8,9]. To date, the degree of conversion after surface finishing has not been investigated, although this is clinically important as the restoration is routinely finished by polishing or grinding.

For the successful and long-term luting of additively manufactured resin-based restorations, the bond strength of the bonding area can be considerably increased by mechanical pretreatment with aluminum oxide (Al_2_O_3_) particles, as the surface area is enlarged, and micromechanical retention promotes the wetting of the luting composite [10]. Additional conditioning with adhesive systems enables the chemical bond to the organic resin matrix in both the object and the luting composite. Progress in the field of the different adhesive systems has allowed the successful implementation of universal adhesives, which are particularly easy and variable to use. Investigations on the bonding properties of universal adhesives employed using self-etch mode or with an additional phosphoric acid etching on enamel and dentin have been carried out [11,12]. Functional acid-modified methylmethacrylates, such as 10-methacryloyloxydecyl dihydrogen phosphate (10-MDP), and silanes provide a chemical bond to zirconia [13], glass-ceramics [14], metals [15], and composites [16]. The chemical structure of the adhesives is equally important [17], as the increased cross-linking and the formation of hydrogen bonds increase its stability and thus its bonding performance [18,19].

New materials such as 3D printable resins are increasingly being introduced to the dental market by manufacturers and the indications are steadily increasing. However, especially when used as fixed prostheses, adhesive luting is a decisive factor for long-term success [20,21]. To the best of the authors’ knowledge, the influence of the cleaning protocol of the printed objects in combination with the application of differently chemically structured adhesive systems and an additional artificial aging on the bond strength between a 3D printed resin and a luting composite resin has not yet been investigated. Therefore, the aim of this investigation was to examine the degree of conversion (DC) and the tensile bond strength (TBS) between a 3D printed resin and a conventional resin-based luting composite following various cleaning procedures and the application of different adhesive systems after varying aging regimens. The first null hypothesis stated that neither the choice of cleaning protocol, nor the use of different adhesive systems, nor the aging regimen show an impact on TBS. The second null hypothesis was that the different cleaning procedures show no impact on the degree of conversion before and after air abrasion.

## 2. Materials and Methods

**Figure 1:**
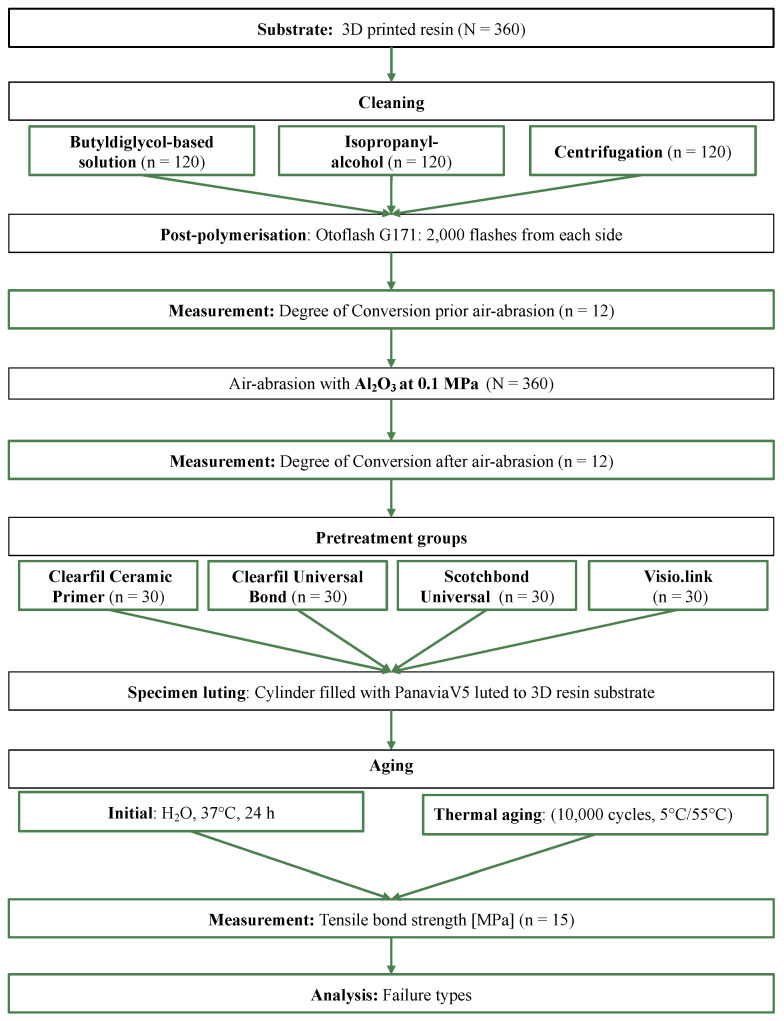
Study design.

Square specimens (4 × 15 × 15 mm^3^) were fabricated using a CAD software (Autodesk Netfabb Basic 2022.0, San Rafael, CA, USA) and exported as STL file. A total of 360 specimens (printo dent Generative Resin GR-17.1 temporary lt, Pro3dure medical, Iserlohn, Germany) were aligned vertically to the printer building platform and additively manufactured with a layer thickness of 50 µm using a 3D printer with digital light processing (DLP) technology (D20 II, Rapid Shape, Heimsheim, Germany). An overview of the study design is shown in Figure 1.

Directly after 3D printing, the specimens were cleaned to remove remaining unpolymerized monomers, either chemically with butyldiglycol-based solution (BUT) or 100% isopropanol (ISO), or mechanically by centrifugation (CEN). Specimens (*n* = 120) cleaned with BUT (InovaPrint wash, hpdent GmbH; Gottmadingen, Germany) were ultrasonically (Sonorex Super RK 102H, Bandelin, Berlin, Germany) cleaned for 2 min according to manufacturer’s recommendation, whereas specimens (*n* = 120) cleaned with ISO (SAV LP, Flintsbach Germany) were activated for 4 min in an ultrasonic bath. Specimens were dried using compressed air. For the mechanical cleaning method, the specimens (*n* = 120) were individually positioned into centrifugal tubes (Polypropylene Conial Tube, BD Falcon, Franklin Lakes, NJ, USA) and centrifugated at 600 G for 10 min (Allegra X-15R, Beckmann Coulter GmbH, Krefeld, Germany). The specimens were then post-cured (Otoflash G171, NK Optik, Baierbrunn, Germany) from both sides with 2000 light flashes in the wavelength range of 280–580 nm under a nitrogen atmosphere to prevent oxygen inhibition on the surfaces.

After storage in distilled water (37 °C) for 24 h in an incubator (HeraCell 150, Heraeus, Hanau, Germany) the bonding areas of the specimens were air-abraded (basis Quattro IS, Renfert GmbH, Hilzingen, Germany) with Al_2_O_3_ (Orbis Dental Handelsgesellschaft mbH, Münster, Germany) with a mean particle size of 50 µm at 0.1 MPa pressure (10 s, 45°, 10 mm distance) followed by cleaning for 3 min in an ultrasonic bath.

Thereafter, the specimens were randomly divided into groups with pretreatment of the bonding area as follows (*n* = 30):

1. Clearfil Ceramic Primer Plus [CCP] (Kuraray Noritake, Okayama, Japan):
  The primer was applied in a thin layer with a microbrush and waited for 20 s.
2. Clearfil Universal Bond Quick [CUP] (Kuraray Noritake Okayama, Japan):
  The universal adhesive was mixed 1:1 with Clearfil DC-Activator (Kuraray Noritake Okayama, Japan), then applied with a microbrush, and subsequently air dried for 5 s.
3. Scotchbond Universal Plus [SUP] (3M, Saint Paul, MN, USA):
  The universal adhesive was applied, massaged for 20 s with a microbrush, and then air dried for 5 s.
4. Visio.link [VL] (Bredent, Senden, Germany):
  The resin primer was applied with a microbrush, then light cured for 90 s with a manufacturer-recommended light-curing unit (bre.Lux Power unit, bredent, Senden, Germany).

The compositions of the used materials are presented in Table 1. Acrylic cylinders (SD Mechatronik, Feldkrichen-Westerham, Germany) with an inner diameter of 2.9 mm were filled with dual adhesive-curing resin-based composite (Panavia V5, Kuraray Noritake, Okayama, Japan) via automix syringe, placed on the bonding area, and light-cured (Elipar Deep Cure-S, 3M, Seefeld, Germany) at room temperature (23 °C, 60% humidity) for a total of 40 s (10 s from the three different sides and 10 s from the top).

All specimens were stored for 24 h in distilled water (37 °C). Half of the specimens were then thermocycled (SD Mechatronik, Feldkirchen-Westerham, Germany) with 10,000 cycles between 5 and 55 °C, each with a drip-off time of 5 s, remaining in each bath for 20 s.

**Figure 2:**
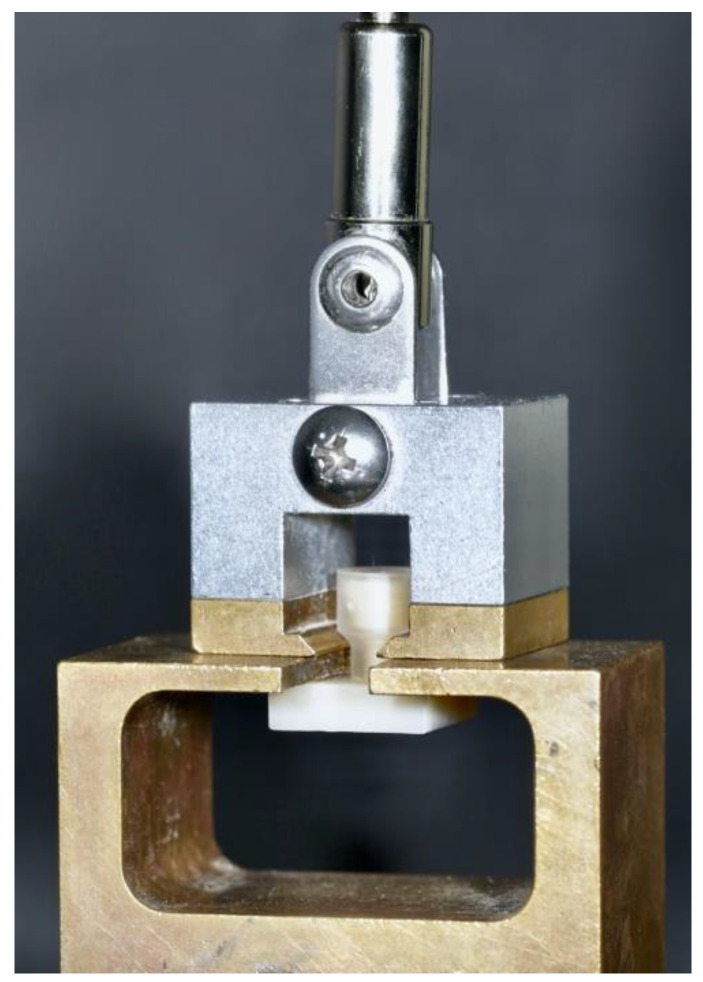
TBS method.

TBS measurements (Figure 2) were performed at room temperature (22 °C, 60% humidity) using a universal testing machine (Zwick 1445, Zwick, Ulm, Germany). The bonded acrylic cylinders were passively fixed into a holding device and pulled with a crosshead speed of 5 mm/min until debonding occurred. The force was applied perpendicular to the bonding area.

TBS was determined according to the following equation, where s is the TBS [MPa], F is the load at fracture [N], and A is the bonding area [mm^2^]:

$$s=\frac{F}{A}$$

**Figure 3:**
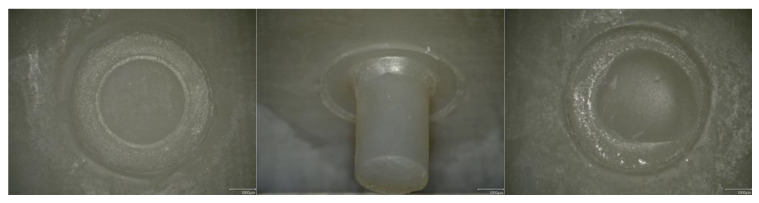
Overview of failure types: Adhesive between the substrate and the luting composite (**left**), cohesive within the luting composite (**middle**), and cohesive within the 3D printed resin (**right**).

The failure types were analyzed under a digital microscope with a 30× magnification (VHX-970F, Keyence, Osaka, Japan) and classified as follows: (i) adhesive between the substrate and the luting composite, (ii) cohesive within the luting composite, and (iii) cohesive within the 3D printed resin (Figure 3).

The degree of conversion (DC) of the three differently cleaned groups were determined using Raman spectra recorded on a confocal Raman spectrophotometer (inVia Qontor, Renishaw, New Mills, UK). Twelve specimens of non-polymerized 3D resin, spread on a microscope slide, and twelve specimens per group, examined directly after post-polymerization and after air-abrasion, were exposed to a high-power near infra-red (HPNIR) laser at a wavelength of 785 nm and a spectral resolution of 1 cm^−1^ through a microscope objective (50×). Each measurement was completed with five accumulations at a laser power of 100% and an irradiation time of 5 s. The recorded spectra were edited in the software WiRE 4.2 (Renishaw, New Mills, UK) in a spectral region of 1500–2000 cm^−1^, including the removal of a baseline, fitting of the determined curves, and the determination of the height of the different peaks. The peak values at 1640 cm^−1^ and 1610 cm^−1^ were analyzed. The degree of conversion (DC) was calculated with the following equation:

$$DC\left(\%\right)=100\times \left(1-\frac{{\left(1640{cm}^{-1}/1610{cm}^{-1}\right)}_{polymerized}}{{\left(1640{cm}^{-1}/1610{cm}^{-1}\right)}_{unpolymerized}}\right)$$

A statistical evaluation of the data were performed using descriptive analysis followed by Kolmogorov–Smirnov to test the violation of normal distribution. Parametric tests were performed, as all groups were normally distributed. To determine the influence of the cleaning methods and pretreatment on TBS, one-way ANOVA with partial eta-squared (η_P_^2^) followed by Scheffé post-hoc test was computed. A two-group *t*-test investigated the impact of the aging regimen. The data were analyzed with SPSS version 26.0 (IBM, SPSS, Statistics, Armonk, NY, USA). The frequency of fracture types was analyzed by chi-square test and Ciba-Geigy table. Statistical significance was inferred when *p*-values < 0.05.

## 3. Results

The results of the descriptive analyses are presented in Table 2 and Table 3.

### 3.1. Degree of Conversion

Directly after post-polymerization, CEN showed lower DC values than BUT and ISO (*p* < 0.001). After air abrasion, BUT showed higher DC values compared to CEN (*p* = 0.024). CEN presented a higher DC after air abrasion (*p* < 0.001) than prior to air abrasion, whereas ISO showed lower DC values after air abrasion (*p* = 0.016).

### 3.2. Tensile Bond Strength

The highest impact on TBS was exerted by the pretreatment method (η_P_^2^ = 0.497, *p* < 0.001), followed by the cleaning procedure (η_P_^2^ = 0.104, *p* < 0.001), and aging (η_P_^2^ = 0.015, *p* = 0.026). Furthermore, the effect of the combination of the three parameters was significant for cleaning coupled with pretreatment methods (η_P_^2^ = 0.155, *p* < 0.001) and for pretreatment methods coupled with aging (η_P_^2^ = 0.088, *p* < 0.001).

Regarding the pretreatment methods, CCP presented the lowest TBS values (*p* < 0.001–0.012) for groups cleaned with BUT or ISO. Pretreatment with CUB (*p* < 0.001–0.034) or SUP (*p* < 0.001–0.023) showed initially higher values than CCP and VL. Pretreatment with SUP led to higher values than CUB (*p* = 0.048) for artificially aged BUT-cleaned specimens. VL presented lower values compared to SUP (*p* = 0.027) for thermocycled ISO-cleaned specimens. No impact of the pretreatment method (*p* = 0.703–0.998) on TBS could be observed for aged, centrifuged groups.

Regarding the cleaning methods, CEN led to higher values for groups pretreated with CCP (*p* < 0.001). Cleaning with BUT initially showed lower values (*p* = 0.024) compared to ISO, when pretreated with VL.

**Figure 4:**
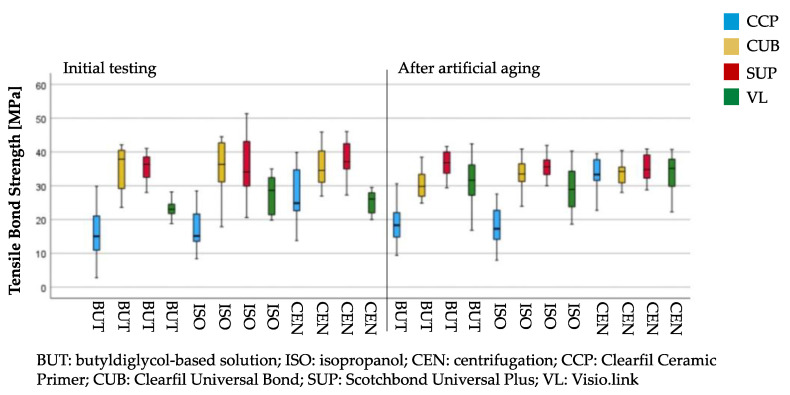
Tensile bond strength values of all tested groups. Colors encode the four conditioning methods.

Regarding the aging regime, thermocycling increased TBS values for groups cleaned with CEN and pretreated with CCP (*p* = 0.013) or VL (*p* < 0.001). Higher values were observed within initial measurements for specimens cleaned with BUT and pretreated with CUB (*p* = 0.025). Thermocycled specimens cleaned with BUT and pretreated with VL (*p* = 0.001) showed higher values than at the initial state (Figure 4).

### 3.3. Failure Types

Groups conditioned with CUB, SUP, and VL showed predominantly cohesive failures within the luting composite resin (27–80%) or cohesive failures within the 3D printed resin substrates (20–73%) (Table 4). For groups conditioned with CCP, mostly adhesive failures occurred (7–80%).

## 4. Discussion

A reliable luting strategy for definitive restorations fabricated with novel 3D printing resin materials coupled with considerations of the highest possible biocompatibility is an important factor in fixed prosthetics. The aim of this investigation was to examine the DC proximately after post-processing of the 3D printed substrates, and the TBS between a 3D printed resin and a conventional luting composite resin following various cleaning procedures and the application of different adhesive systems after varying aging regimen. The present investigation showed that the type of cleaning, the choice of adhesive system, and the aging affect the TBS. Likewise, the measured degree of conversion showed differences according to the chemical and mechanical cleaning method. Thus, the first and the second tested null hypotheses could be rejected.

After centrifugation, a sticky surface with a visible amount of non-polymerized resin remained [4]; this was evident from a shiny surface after post-polymerization with Otoflash G171 in a nitrogen atmosphere. Since Raman spectra measure point values on the surface, it may lead to the conclusion that Otoflash G171 is not able to cure the resin as sufficiently as when polymerization is performed during the 3D printing process. After removing the shiny surface by air abrasion, the Raman spectra might measure deeper areas, resulting in comparable DC rates to chemical cleaning [8]. With the surface layers of the restoration being routinely removed by finishing and polishing, the type of cleaning may, however, play a subordinate role in biocompatibility.

Cleaning with isopropanol, on the other hand, showed a higher carbon conversion rate after post-polymerization than after sandblasting. This finding may be explained by the effective removal of the adherent liquid resin off the surface and a deterioration of the DC rate in deeper layers [22].

Air abrasion with Al_2_O_3_ particles exhibited good results in tensile [23,24] and shear bond strength [25] to a luting composite resin in both subtractively and additively manufactured restorations. All groups were mechanically air-abraded with Al_2_O_3_ particles before conditioning with adhesives, which made the results comparable.

The tested adhesive systems differ in their composition. The highest TBS values were achieved by pretreatment of the luting area with universal adhesives i.e., CUB and SUP. The universal adhesives used contain acid-modified monomers with bifunctional properties. Acidic phosphoric monomers (10-MDP) interact ionically with the oxide ceramic fillers in the restoration and additionally enable the bonding of Ca^2+^ ions in the tooth structure [26]. In addition, on one side, silane-reactive hydroxyl groups form a covalent bond with the glass-ceramic fillers through a condensation reaction and, on the other side, regular organophilic methacrylate groups can copolymerize with the luting composite resin [27],

In contrast to the universal adhesives, the 10-MDP silane primer, CCP, achieved lower TBS values. In addition, more adhesive failures were observed, although none of the specimens pretreated with CCP debonded prematurely. Reasons for this could be the chemical composition of the silane primer and the 3D printable resin. The silane primer bonds to glass–ceramic fillers, but at the same time blocks the formation of carbon bonds between the 3D resin and the luting composite resin [28].

Due to its low viscosity, which is important for processing in the printer, the 3D resin has the characteristics of a flowable composite [29] and consists mainly of the resin matrix (60%) and a reduced number of fillers (40%) according to manufacturers’ specifications.

Interestingly, cleaning by centrifugation can improve the TBS values of the CCP pretreated specimens. This could be due to the mechanical cleaning, as no ingredients are dissolved in the 3D printing resin, as is the case of the chemical cleaning. With the chemical cleaning, it would be conceivable that, in addition to residual monomers, glass–ceramic fillers are also dissolved from the 3D resin matrix and are thus no longer available for chemical bonding. Visio.link containing methylmethacrylate (MMA) performed slightly better than the silane primer. MMA attacks the top layer and dissolves existing double bonds, promoting the bond to the matrix, especially for materials containing polymethylmethacrylate (PMMA) [30]. A swollen and dissolved material after application of MMA monomers to the surface of 3D printed temporary substrates has already been reported [31]. Nevertheless, no chemical interaction with the fillers is generated.

In the present investigation, artificial aging was carried out with 10,000 thermal cycles to simulate a period in the oral cavity at normal daily temperature changes (e.i. eating, drinking, and breathing). It is possible to expect that, especially after centrifugation, the increased proportion of remaining free carbon–carbon double bonds at warmer temperatures (55 °C) will promote additional co-polymerization with the luting composite resin [32]. While it can also be assumed that the aging process promotes the co-polymerization of Visio.link, it remains unclear how the cleaning method influences polymerization. On the other hand, hydrolytic degeneration and high temperature variations (5/55°) to which the specimens are exposed could increase the coefficient of thermal expansion at the bonding interface, which could lead to cracks and result in lower TBS values [33]. However, a decrease in TBS values could only be observed in one of the tested groups (BUT-cleaned specimens pretreated with CUB). A variety of in vitro bond strength tests can evaluate the quality of adhesion. In the present study, macro tensile tests were performed as they proved to be more clinically relevant compared to shear bond strength tests, as they often indicate cohesive failures, and therefore it is assumed that this method measures not only bond strength but also overall stability [34]. In addition, macro tensile strength tests offer advantages over micro tensile strength tests, as they allow a specimen preparation without additional mechanical pre-stressing [35].

According to the TBS measurements, only cohesive failures were observed after pretreatment with CUB, SUP, and VL. It can be assumed that the bond strength is stronger than the overall stability of the 3D printed substrates or the luting composite resin. In contrast, adhesive failures of up to 80% were observed when pretreating with CCP. Thus, it can be considered that the tensile test provided a correct measurement of the bond strength.

A limitation of the present study is that a power analysis was conducted post-hoc. The post-hoc power analysis on a specimen number of 15 specimens showed that the resulting power of a two-sided *t*-test comparing the results of specimens conditioned with Visio.link and pretreated with ceramic primer and measured in the initial state is 99%, with an effect of 10.36 MPa and a pooled standard deviation of 6.22 MPa. A second post-hoc analysis was performed with the same groups (specimens conditioned with Visio.link compared to pretreatment with ceramic primer) only after artificial aging and showed that the resulting power of a two-sided *t*-test is 99%, given a sample size of 15, with an observed effect of 11.45 MPa and a pooled standard deviation of 6.76 MPa. On the one hand, the two groups were selected from the isopropanol cleaning group, as this cleaning is most frequently used for cleaning 3D printed objects and is recommended by most manufacturers. On the other hand, conditioning with Visio.link or silane coupling agents (CCP) are among the most practically relevant pretreatment methods in the dental laboratory and in practice [36,37,38,39].

The physical cleaning of fixed dental protheses by means of centrifugal force showed a comparable bond strength among the adhesives tested, especially for the aged specimens. This was in contrast to the chemical cleaning, which showed clear differences in the choice of the adhesive. The results of this study should be interpreted with caution as the in vitro design does not reflect all clinically relevant factors. In vivo studies are needed to evaluate the bond strength of additively manufactured restorations.

## Figures and Tables

**Table 1:** Summary of the adhesive and luting materials, compositions, LOT-Numbers, and expiration.

	Material	Abbreviation	Composition	Manufacturer	LOT	Expiry
Conditioning method	Clearfil Ceramic Primer Plus	CCP	Ethanol, 3-Methacryloyloxypropyltrimethoxysilan, 10-MDP ^a^	Kuraray Noritake Okayama, Japan	5D0063	29.2.24
	Clearfil Universal Bond Quick	CUB	Bisphenol A diglycidylmethacrylat, ethanol, 2-hydroxyethylmethacrylat, 10-MDP, hydrophilic amide monomers, colloidal silica, silane, water ^b^	Kuraray Noritake Okayama, Japan	4N0301	30.9.24
	Scotchbond Universal Plus	SUP	MDP, Vitrebond-Copolymer, silica fillers, ethanol, water, initiators, amino functional silane, dimethacrylate (bisphenol A-free), pH = 2.7 ^c^	3M, Seefeld, Germany	7172629	30.4.24
	Visio.link	VL	MMA, 2-Propenoic acid, bisphenol-A diglycidyl-methacrylate, diphenyl(2,4,6-trimethylbenzyl) phosphinoxidec ^d^	Bredent, Senden, Germany	193211	31.8.24
Resin-based composite	Panavia V5		Bisphenol-A-diglycidylmethacrylat, triethylenegycol-dimethacrylat, titanoxide, colloidal silica, silanised barium glass filler, silanised fluoroaluminosilicate, alumina filler, hydrophobic aromatic dimethylacrylate, aliphatic dimethylacrylate, initiatiors, pigments ^e^	Kuraray Noritake Okayama, Japan	760165	30.4.24
3D printable resin	printo dent Generative Resin GR-17.1 temporary lt		Methacrylic resins < 60% (mainly Bisphenol-A ethoxylate dimethacrylate), metal oxides, photoinitiators < 2% (mainly TiO_2_, TPO), UV inhibitors < 0.1%, inorganic glass fillers 40% ^f^	Pro3dure medical, Iserlohn, Germany	03082017	03.8.23

^a^ Kuraray Noritake, Clearfil Ceramic Primer Plus, safety data sheet, 2015, status 23 October 2022. ^b^ Kuraray Noritake, Clearfil Universal Bond Quick, safety data sheet, 2021, status 23 October 2022. ^c^ 3M, Scotchbond Universal Plus Adhesive, Technical Product Profile, 2021, status 23 October 2022. ^d^ bredent, Visio.Link, safety data sheet, 2020, status 23 October 2022. ^e^ Kuraray Noritake, Panavia V5, safety data sheet, 2021, status 23 October 2022. ^f^ Pro3dure medical manufacturer information, status 05 April 2022.

**Table 2:** DC for all tested cleaning groups with descriptive statistics (Mean ± SD, Min/Med/Max) and 95% confidence intervals (CI).

Cleaning	Mean ± SD	95% CI	Min/Med/Max
Prior to air abrasion			
BUT	96.6 ± 0.9 ^bA^	(95.9/97.1)	95.2/96.8/97.7
ISO	95.5 ± 0.7 ^bB^	(96.0/96.9)	95.4/96.3/97.9
CEN	88.4 ± 0.7 ^aA^	(87.8/88.8)	87.3/88.5/89.3
After air abrasion			
BUT	95.1 ± 1.4 ^bA^	(94.2/95.9)	92.6/95.4/96.9
ISO	94.4 ± 2.6 ^abA^	(92.7/96.0)	89.7/94.6/98.5
CEN	92.7 ± 1.9 ^aB^	(91.4/93.9)	90.1/93.0/95.4

^ab^: different letters indicate significant differences between cleaning groups within one pretreatment group (prior to air abrasion or after air abrasion). ^AB^: different letters indicate significant differences between the pretreatment groups within one cleaning group.

**Table 3:** Descriptive statistics with mean and standard deviation (SD), the minimum/median/maximum (Min/Med/Max) and 95% confidence intervals (CI) for TBS in MPa per cleaning, pretreatment, and aging group.

	BUT			ISO			CEN		
	Mean ± SD	95% CI	Min/Med/Max	Mean ± SD	95% CI	Min/Med/Max	Mean ± SD	95% CI	Min/Med/Max
Pretreatment	Initial								
CCP	16 ± 7 ^aAi^	(11; 21)	3/15/30	17 ± 6 ^aAi^	(12; 21)	8/43/59	27 ± 8 ^aBi^	(21; 32)	14/25/40
CUB	40 ± 5 ^cAii^	(36; 44)	28/41/48	36 ± 8 ^cAi^	(30; 41)	18/36/45	36 ± 6 ^bAi^	(31; 40)	27/35/46
SUP	36 ± 4 ^cAi^	(33; 38)	28/36/41	36 ± 9 ^cAi^	(30; 42)	21/34/51	38 ± 5 ^bAi^	(34; 41)	27/37/46
VL	24 ± 3 ^bAi^	(20; 25)	19/23/30	27 ± 6 ^bBi^	(26; 43)	20/29/35	25 ± 3 ^aABi^	(22; 27)	20/26/30
	Artificial aging								
CCP	19 ± 7 ^aAi^	(14; 23)	9/18/31	18 ± 6 ^aAi^	(13; 21)	8/15/28	33 ± 5 ^aBii^	(29; 37)	23/33/40
CUB	33 ± 4 ^bAi^	(27; 33)	25/30/38	34 ± 5 ^bcAi^	(30; 37)	24/33/41	34 ± 4 ^aAi^	(30; 36)	28/34/40
SUP	36 ± 4 ^cAi^	(32; 39)	29/37/42	36 ± 4 ^cAi^	(32; 38)	30/36/42	35 ± 4 ^aAi^	(33; 37)	29/35/41
VL	31 ± 7 ^bcAii^	(27; 35)	17/32/42	29 ± 7 ^bAi^	(24; 33)	19/29/40	34 ± 6 ^aAii^	(29; 38)	22/36/41

BUT: butyldiglycol-based solution; ISO: isopropanol; CEN: centrifugation; CCP: Clearfil Ceramic Primer; CUB: Clearfil Universal Bond; SUP: Scotchbond Universal Plus; VL: Visio.link. ^abc^: different lowercase letters indicate significant differences between the pretreatment methods within one cleaning and the aging group. ^AB^: different uppercase letters indicate significant differences between the cleaning methods within one pretreatment and the aging group. ^i,ii^: different letters indicate significant differences between the aging regimen within one cleaning and the pretreatment group.

**Table 4:** Percentage of evaluated failure types and 95% CI for TBS [MPa] per cleaning, pretreatment, and aging group.

Initial		Adhesive Failures (%) and 95% CI	Cohesive Failures within Luting Resin (%) and 95% CI	Cohesive Failures within 3D Resin (%) and 95% CI
Cleaning	Pretreatment			
BUT				
	CCP	73 (43; 93)	0 (0; 22)	27 (6; 56)
	CUB	0 (0; 22)	67 (37; 89)	33 (10; 62)
	SUP	0 (0; 22)	40 (15; 68)	60 (31; 84)
	VL	0 (0; 22)	33 (10; 62)	67 (37; 89)
ISO				
	CCP	87 (58; 99)	0 (0; 22)	13 (0; 41)
	CUB	0 (0; 22)	47 (20; 74)	53 (25; 79)
	SUP	0 (0; 22)	53 (25; 79)	47 (20; 74)
	VL	0 (0; 22)	67 (37; 89)	33 (10; 62)
CEN				
	CCP	20 (3; 47)	7 (0; 32)	73 (43; 93)
	CUB	0 (0; 22)	47 (20; 74)	53 (25; 79)
	SUP	0 (0; 22)	73 (43; 93)	27 (6; 56)
	VL	0 (0; 22)	27 (6; 56)	73 (43; 93)
**Artificial** **Aging**		**Adhesive Failures** **(%) and 95% CI**	**Cohesive Failures within Luting Resin (%) and 95% CI**	**Cohesive Failures within 3D Resin (%) and 95% CI**
Cleaning	Pretreatment			
BUT				
	CCP	33 (10; 62)	7 (0; 32)	60 (31; 84)
	CUB	0 (0; 22)	60 (31; 84)	40 (15; 68)
	SUP	0 (0; 22)	67 (37; 89)	33 (10; 62)
	VL	0 (0; 22)	53 (25; 79)	47 (20; 74)
ISO				
	CCP	67 (37; 89)	0 (0; 22)	33 (10; 62)
	CUB	0 (0; 22)	47 (20; 74)	53 (25; 79)
	SUP	0 (0; 22)	53 (25; 79)	47 (20; 74)
	VL	0 (0; 22)	53 (25; 79)	47 (20; 74)
CEN				
	CCP	7 (0; 32)	53 (25; 79)	40 (15; 68)
	CUB	0 (0; 22)	60 (31; 84)	40 (15; 68)
	SUP	0 (0; 22)	67 (37; 89)	33 (10; 62)
	VL	0 (0; 22)	80 (50; 96)	20 (3; 47)

## Data Availability

On reasonable request, the corresponding author will make the datasets generated during the current investigation available.
